# Mesenchymal Stem Cells as Therapeutic Candidates for Halting the Progression of Diabetic Nephropathy

**DOI:** 10.1155/2016/9521629

**Published:** 2016-12-13

**Authors:** Janaina Paulini, Eliza Higuti, Rosana M. C. Bastos, Samirah A. Gomes, Érika B. Rangel

**Affiliations:** ^1^Sociedade Beneficente Albert Einstein, Albert Einstein Hospital, 05652 São Paulo, SP, Brazil; ^2^University of São Paulo, 01246 São Paulo, SP, Brazil; ^3^Federal University of São Paulo, 04023 São Paulo, SP, Brazil

## Abstract

Mesenchymal stem cells (MSCs) possess pleiotropic properties that include immunomodulation, inhibition of apoptosis, fibrosis and oxidative stress, secretion of trophic factors, and enhancement of angiogenesis. These properties provide a broad spectrum for their potential in a wide range of injuries and diseases, including diabetic nephropathy (DN). MSCs are characterized by adherence to plastic, expression of the surface molecules CD73, CD90, and CD105 in the absence of CD34, CD45, HLA-DR, and CD14 or CD11b and CD79a or CD19 surface molecules, and multidifferentiation capacity in vitro. MSCs can be derived from many tissue sources, consistent with their broad, possibly ubiquitous distribution. This article reviews the existing literature and knowledge of MSC therapy in DN, as well as the most appropriate rodent models to verify the therapeutic potential of MSCs in DN setting. Some preclinical relevant studies are highlighted and new perspectives of combined therapies for decreasing DN progression are discussed. Hence, improved comprehension and interpretation of experimental data will accelerate the progress towards clinical trials that should assess the feasibility and safety of this therapeutic approach in humans. Therefore, MSC-based therapies may bring substantial benefit for patients suffering from DN.

## 1. Introduction

Diabetes mellitus (DM) is a global epidemic disease that affects people of all ages, gender, and ethnicity. The prevalence of DM for all age-groups was estimated to be 2.8% in 2000 and 4.3% in 2030. The total number of people with DM is projected to rise from 171 million in 2000 to 366 million in 2030 according to World Health Organization [1].

Diabetic nephropathy (DN) is the leading cause of chronic kidney disease in patients starting renal replacement therapy, affecting ~40% of type 1 and type 2 diabetic patients [2]. DN is defined by increased urinary albumin excretion (UAE) in the absence of other renal diseases and is categorized into the following stages: microalbuminuria (UAE 20 *μ*g/min–199 *μ*g/min or 30–299 mg/24 h) and macroalbuminuria (UAE ≥ 200 *μ*g/min or ≥300 mg/24 h) [3]. In patients with type 2 diabetes, the incidence of microalbuminuria is 2% per year and the prevalence at 10 years after diagnosis is 25% [4]. Proteinuria occurs in 15–40% of patients with type 1 diabetes, with a peak in 15–20 years after DM diagnosis [5]. Likewise, in patients with type 2 diabetes, the prevalence of proteinuria is variable, ranging from 5 to 20% [4]. The stage of DN has a positive association with increased mortality of all causes and from cardiovascular, cerebrovascular, and coronary heart diseases [4, 6].

Clinical manifestations of DN, such as proteinuria, increased blood pressure, and decreased glomerular filtration rate are similar in type 1 and type 2 diabetes and correlate strongly with structural abnormalities. Morphologically, DN is characterized by thickening of the glomerular basement membrane (GBM) and mesangial expansion, leading to a progressive reduction in the filtration surface of the glomerulus [7]. Although the most important structural changes occur in the glomeruli, concomitantly and approximately in proportion to the degree of glomerulopathy, abnormalities in the tubule-interstitial and arteriolar compartments contribute to the pathogenesis of DN [7, 8].

Despite the fact that pancreas transplant may reverse the thickness of the glomerular and tubular basement membranes after five years of normoglycemia [9], that procedure is associated with adverse effects of immunosuppressive regimen [10] and immunological risk that affect long-term survival [11].

DN progression may be prevented by tight glucose control, blood pressure control, renin-angiotensin-aldosterone system (RAAS) blockade, smoking cessation, weight loss, and physical activity [3].

To note, mesenchymal stem cell- (MSC-) based therapies have been expected to bring substantial benefit to patients suffering a wide range of diseases and injuries. This article reviews the existing literature and knowledge of MSC therapy in DN and highlights some preclinical relevant studies and new perspectives of combined therapies for decreasing DN progression.

## 2. The Pathogenesis of Diabetic Nephropathy (DN)

DN is initially characterized by functional glomerular changes, including glomerular hyperperfusion and hyperfiltration, before the onset of any measurable clinical changes. As DN evolves, thickening of the GBM, glomerular hypertrophy, and mesangial expansion take place.

Despite the fact that several factors have been implicated in the pathogenesis of DN, we will focus on the particular factors outlined above [12–17]:

### 2.1. Hemodynamics Pathways

The early signs of glomerular hyperperfusion and hyperfiltration result from decreased resistance of renal arterioles (afferent > efferent) which are mediated by prostanoids, nitric oxide, vascular endothelial growth factor A (VEGF-A), transforming growth factor-*β*1 (TGF-*β*1), endothelin, and RAAS. These effects facilitate localized release of certain cytokines and growth factors, leading ultimately to albumin leakage from the glomerulus and structural changes, for example, overproduction of mesangial cell matrix, thickening of GBM, and podocyte damage.

### 2.2. Hyperglycemia and Advanced Glycosylation End Products

Hyperglycemia is a key factor in developing DN due to its effect on mesangial cell proliferation, hypertrophy, and apoptosis, as well as an increased matrix production and GBM thickening. These effects are mediated by upregulation of glucose transporters (GLUT1 and GLUT4) and an increase in glucose entrance into the cells.

Hyperglycemia mediates tissue damage by inducing nonenzymatic glycosylation that generates advanced glycosylation end products (AGEs; the cross-link with collagen I contributes to microvascular complications), activation of protein kinase C (PKC; activates vasodilatory prostanoids which contributes to glomerular hyperfiltration, as well as TGF-*β*1), and acceleration of the aldose reductase pathway. All these three pathways are related to oxidative stress.

### 2.3. Cytokines

Activation of cytokines, profibrotic and inflammatory elements, and vascular growth factors might be involved in the matrix accumulation that takes place in DN. VEGF and angiopoetin contribute to retinopathy, although their effects on DN are not conclusive. To note, VEGF may increase permeability of the glomerular filtration barrier to proteins. Hyperglycemia, TGF-*β*1, and angiotensin II stimulate VEGF expression, which ultimately leads to the production of endothelial nitric oxide and *α*3 chain of collagen IV. On the other hand, VEGF might be a crucial factor secreted by podocytes to maintain both glomerular endothelial cell and mesangial cell proliferation and differentiation.

TGF-*β*1 contributes to cell hypertrophy and increased synthesis of collagen, leading ultimately to glomerulosclerosis and tubule-interstitial injury during DN development. Hepatocyte growth factor (HGF) ameliorates DN by blocking the profibrotic actions of TGF-*β*1.

Inflammatory cytokines also contribute to the development and progression of DN, mainly interleukin-1 (IL-1), IL-6, IL-18, and tumor necrosis factor-*α* (TNF-*α*). Each cytokine possesses different effects. IL-1 alters the expression of chemotactic factors and adhesion molecules, alters intraglomerular hemodynamics mediated by prostaglandins, increases vascular endothelial cell permeability, and increases hyaluronan production by renal tubular epithelial cells. IL-6 promotes GBM thickening, endothelial permeability, and mesangial expansion. IL-18 is associated with endothelial cell apoptosis and secretion of other inflammatory cytokines (IL-1, interferon *γ* [INF-*γ*], and TNF-*α*). TNF-*α* affects apoptosis, glomerular hemodynamics, endothelial permeability, and cell-cell adhesion.

### 2.4. Lipid Mediators

Prostaglandin E2 and I2 might promote renal inflammation and renal inhibition of cyclooxygenase 2 is associated with a decrease in glomerular hyperfiltration. Lipoxygenases 12 and 15 are also increased in DN. Furthermore, arachidonic acid oxidation might be related to mesangial cell hypertrophy and extracellular matrix accumulation by TGF-*β*1 and angiotensin II.

### 2.5. Oxidative Stress

Reactive oxygen species, generated in the mitochondria, mediate many negative biological effects, including peroxidation of cell membrane lipids, oxidation of proteins, renal vasoconstriction, and damage to DNA, as well as PKC activation and AGEs formation.

### 2.6. Genetic Susceptibility

In patients with type 1 and type 2 diabetes, the likelihood of developing DN is increased in those who have a sibling or parent with DN. Genotyping single-nucleotide polymorphism investigation indicates that some* loci* are identified as DN susceptibility genes areas on chromosomes 7q21.3, 10p15.3, 14q23.1, and 18q22.3. Likewise, association studies of candidate genes suggested that the angiotensin-converting-enzyme gene (ACE) DD polymorphism might be associated with increased risk of developing DN in type 2 diabetic patients. However, identification of a multigene panel seems to be the most appropriated approach to study the susceptibility to DN.

## 3. Mesenchymal Stem Cells (MSCs)

MSCs, commonly referred to as mesenchymal stem cells or mesenchymal stromal cells, are a diverse population of cells with a wide range of potential therapeutic applications for different organs and tissues. MSCs can be derived from many tissue sources, consistent with their broad, possibly ubiquitous distribution.

Stem cells are characterized by their ability to self-renew, clone, differentiate into different lineages, and regenerate damaged organ. The International Society for Cell Therapy (ISCT) proposed a criteria to define human (h) MSC that comprises the following: (1) adherence to plastic in standard culture conditions; (2) expression of the surface molecules CD73, CD90, and CD105 in the absence of CD34, CD45, HLA-DR, and CD14 or CD11b and CD79a or CD19 surface molecules, as assessed by flow cytometry analysis; (3) capacity for differentiation to osteoblasts, adipocytes, and chondroblasts in vitro [18]. These criteria were established to standardize human (h) MSC isolation but may not uniformly apply to other species.

Furthermore, murine species obtained from 5 strains were similar to human and rat MSCs in terms of expansion under adherent conditions, single-cell-derived colony formation assay, and multipotent differentiation to osteoblasts, adipocytes, and chondroblasts in vitro [19]. However, the cells from the 5 strains differed in their media requirements for optimal growth, rates of propagation, and presence of the surface epitopes CD34 (B1/6+++, FVB/N++, and BALB/c and DBA1+ and hMSC−), stem cell antigen-1 (Sca-1; B1/6 and FVB/N+++; BALB/c−, DBA1+, and hMSC not available), CD90 (B1/6, FVB/N, and BALB/c and DBA1− and hMSC+++), and vascular cell adhesion molecule 1/CD106 (VCAM-1; B1/6, FVB/N, hMSC+++, and BALB/c and DBA1+). CD45 and CD11b negativity are observed in all murine strains and human MSCs. The differences among MSCs from different strains may explain some of the conflicting data published on the engraftment of mouse MSCs or other bone marrow cells into nonhematopoietic tissues.

### 3.1. Isolation and Sources of MSCs

Historically, MSCs were isolated from bone marrow (BM-MSC) and spleen from guinea pigs by Friedenstein and colleagues [20]. They observed that MSCs were plastic adherent cells and were capable of forming single-cell colonies. When BM-MSCs were expanded in culture, round-shaped colonies resembling fibroblastoid cells formed and were identified by the Colony Forming Unit-fibroblast (CFU-f) assay. They were the first to demonstrate that MSCs exhibited multipotential capacity to differentiate into mesoderm-derived tissues.

MSCs can be isolated (a) by using a gradient centrifugation (Ficoll or Percoll) to separate nonnucleated red blood cells from nucleated cells; (b) by taking advantage of their ability to adhere to plastic; or (c) by the ability of monocytes to be separated from MSCs by trypsinization [21].

During the 1980s, MSCs were shown to differentiate into osteoblasts, chondrocytes, adipocytes, and muscle [22]. In the 1990s, it was documented that MSCs were able to differentiate into ectodermal-derived tissue [23, 24]. During the early 21st century, in vivo studies demonstrated that human MSCs differentiated into endodermal-derived cells [25, 26], cardiomyocytes [27], and renal mesangial and epithelial tubular cells [28, 29]. However, their efficiency to differentiate into other tissues is extremely low in vivo and therefore is not the main mechanism of tissue repair.

MSCs can be isolated from BM, adipose tissue (ADMSC), umbilical cord blood (UCB-MSC), and other tissues. In BM, 1 in 10,000 nucleated cells is a MSC. To note, one gram of aspirated adipose tissue yields approximately 3.5 × 10^5^–1 × 10^6^ ADMSCs. This is compared to 5 × 10^2^–5 × 10^4^ of BM-MSCs isolated from one gram of bone marrow aspirate [30].

MSCs possess ubiquitous distribution in perivascular niches and can be derived and propagated in vitro from different organs and tissues (BM, brain, spleen, liver, kidney, lung, muscle, thymus, pancreas, cord blood, amniotic fluid and placental membranes, and large vessels, such as aorta artery and vena cava) [31, 32].

MSC cell populations originating from different tissues and organs exhibit similar morphology and, to a certain extent, surface marker profile [31]. On the other hand, differentiation assays indicate some variation among cultures in the frequency of cells that possess the capacity to differentiate into osteogenic or adipogenic lineages. For example, vena cava derived MSCs were very efficient at depositing mineralized matrix, whereas muscle-derived MSCs showed little efficiency, although an inverse capacity of adipocyte differentiation was observed with these cells [31]. In contrast, the adipogenic differentiation observed in lung-, brain-, and kidney-derived MSCs seemed to be less efficient. Likewise, UCB-MSCs exhibit significantly stronger osteogenic capacity but lower capacity for adipogenic differentiation in comparison to BM-MSCs [33]. Of importance, ADMSCs exhibit similar capacity of differentiation when compared to BM-MSCs [34].

### 3.2. Paracrine Signaling and Immunomodulatory Effects of MSCs

Notably, the frequency of MSC engraftment and differentiation in different organs is low compared to the robust functional recovery observed after cell transplant, which has raised questions as to whether MSC engraftment and differentiation is the leading mechanism of action. MSCs secrete a wide array of cytokines and growth factors, which can suppress the immune system, fibrosis oxidative stress, and apoptosis and enhance angiogenesis [35].

The effects of MSCs on innate and adaptive immunity have been reported in the literature. MSCs modulate the innate function of monocytes, macrophages, natural killer (NK) cells, and dendritic cells (DCs). They are capable of modifying the maturation of DC, thereby inhibiting their antigen-presenting function an inducing the generation of tolerogenic DCs. Importantly, MSCs show intermediate expression of MHC I (Major Complex of Histocompatibility) and do not express MHC II on their surface, which reduces their antigenicity and increases their tolerability in allogeneic transplant [36–38]. Furthermore, MSCs are capable of suppressing T lymphocytes proliferation and inducing FOXP3^+^CD4^+^ regulatory T lymphocytes (Tregs). Mediators of Treg generation include indoleamine 2,3-dioxygenase (IDO), prostaglandin E2 (PGE2), and IFN-*γ*. In addition, MSCs inhibit the proinflammatory Th17 cell activity.

To note, MSC effect on lymphocytes B cell has been scarcely studied and contradictory, yet it appears that this interaction occurs not only by the modulation of T-helper lymphocyte activity by MSCs, but also by a direct inhibitory mechanism by MSC in B lymphocyte activation [39].

Although BM-MSCs, ADMSCs, and UCB-MSCs equally hamper T lymphocyte, B lymphocyte, and NK cell-mediated immune response by preventing their acquisition of lymphoblast characteristics, activation, and changing the expression profile of proteins with an important role in immune function, UCB-MSCs do not inhibit B cells activation [34, 40].

Although these studies suggest that the use of MSCs in regenerative therapies could be successful, the mechanisms responsible for the tolerance of the host immune system to MSCs are not fully understood. Moreover, all these mechanisms are interrelated and involve both direct cell-cell contact and indirect mechanisms, through the production and release of soluble factors, such as cytokines and hormones.

## 4. Mesenchymal Stem Cells (MSCs) in Diabetic Nephropathy (DN)

### 4.1. MSC Therapy in Small and Large Animals

In 2001, the Animal Models of Diabetic Complications Consortium (AMDCC) was created by National Institutes of Health to develop and characterize models of diabetic nephropathy. Hence, AMDCC defined the following criteria for validating a progressive mouse model of DN [41]: (i) greater than 50% decline in GFR (glomerular filtration ratio) over the lifetime of the animal; (ii) greater than 10-fold increase in albuminuria compared to controls for the strain at the same age and gender; (iii) pathology of kidneys that include advanced mesangial matrix expansion +/− nodular sclerosis and mesangiolysis; any degree of arteriolar hyalinosis; GBM thickening by >50% over baseline, and tubule-interstitial fibrosis.

The most promising strains to study DN, in accordance with AMDCC recommendation, include the following:

(a) eNOS (endothelial nitric oxide synthase) deficient (C57BL/6 and C57BLKS backgrounds) mice: to generate a model of type 1 DM, streptozotocin (STZ) may be injected and to generate a model of type 2 DM; these mice can be crossed with C57BLKS (BKS)-db/db mice
(b) bradykinin B2 receptor deficient (C57BL/6 and C57BLKS backgrounds) mice: these mice can be crossed with *Ins*2^Akita/+^ or BKS-db/db mice to study DN. STZ also promotes DN in these transgenic crossed mice
(c) decorin (inhibitor of TGF-*β*) deficient mice (B6 background)
(d) NONcNZO10/LtJ mice: these mice are derived from a cross between nonobese nondiabetic (NON/lt) strain the New Zealand Obese (NZO/H1Lt) mouse, which provides a model of polygenic type 2 DM)
(e) FVB-OVE26 mice (FVB background): transgenic model of early-onset of type 1 DM.
(f) Renin overexpression (129S6/SvEvTac background): these mice express plasma renin near eight times normal and develop kidney and cardiovascular disease

Although these transgenic mice develop proteinuria and renal histological abnormalities secondary to DN, they do not reliably develop all of the features of human DN. However, two recent models of type 1 and type 2 diabetes that reflect human DN were reported [42, 43]. These models can serve to study the mechanisms that not only lead to the development of DN, but also lead to testing cell therapy, gene therapy, and pharmacologic drugs.

Notably, E1-DN mice were recently described as a model of type 1 diabetes [42]. These mice express a kinase-negative epidermal growth factor receptor in pancreatic islet cells and are diabetic from 2 weeks of age due to impaired postnatal growth of *β*-cell mass. By 10 weeks, they develop proteinuria, mesangial expansion, thickening of GBM, widening of podocyte foot process, podocyte apoptosis, glomerular sclerosis, and reduction of nephrin expression.

The recently described BTBR (black and tan, obese, tufted) ob/ob (leptin deficient) (BTBR^*ob*/*ob*^) mice with type 2 diabetes demonstrate key features of early podocyte loss and mesangiolysis characteristic of human DN [43]. BTBR^*ob*/*ob*^ mice develop progressive proteinuria beginning at 4 weeks. Characteristics of early DN, such as glomerular hypertrophy, reduced podocyte density, and accumulation of mesangial matrix, can be present by 8 weeks. Glomerular lesions similar to those of advanced human DN are present by 20 weeks. By 22 weeks, an approximately 20% increase in basement membrane thickness and a >50% increase in mesangial matrix can be detected and are associated with diffuse mesangial sclerosis (focally approaching nodular glomerulosclerosis); focal arteriolar hyalinosis, mesangiolysis, and focal mild interstitial fibrosis are present.

To note, pharmacologic induction of DN with STZ, with or without accelerating factors, such as high fat diet, uninephrectomy, or use of the nonobese diabetic strain (NOD) strain has been the most common rodent model of DN to study the potential therapeutic of MSCs [35].

Yet pharmacological therapy with angiotensin converting enzyme inhibitors and angiotensin II receptor antagonists, glucose and blood pressure control, and lifestyle modifications [3, 44] are standard-of-care for diabetic individuals; the search for complementary therapeutic approaches to curtail DN progression is required.

MSCs administration is reported to ameliorate renal and pancreatic parameters in terms of dysfunction and morphological abnormalities, as reported in Table 1 [45–60].

MSCs are generally transient cells that exist briefly in the host and cannot be identified after a few days or possibly a week or two. Their safety as allogeneic cell transplants may be closely related to their short-term existence. Their anti-inflammatory properties, homing to sites of damage and inflammation, and their trophic influence on tissue repair have made them a promising strategy for clinical studies.

Although MSC therapy has already been reported to ameliorate kidney and pancreatic injury, many difficulties must be overcome to successfully implement that cell therapy. These difficulties include the definition of the most appropriated route for cell delivery and the number of cells to be injected, the improvement of MSC homing to damaged kidneys, the comprehension of MSC-host cells interaction, and the adverse effects of MSC engraftment (in vivo mal-differentiation and tumor formation).

MSC delivery route is a crucial aspect of cell therapy. As reviewed elsewhere, arterial route for progenitor/stem cell delivery promotes kidney regeneration more efficiently than intravenous route [61]. In intravenous route, the number of cells, multiple intravenous injections, and cell size increase the chance of pulmonary trapping [62, 63]. Although intraparenchymal administration of progenitor/stem cells also has beneficial effect on kidney repair, this route is less practical for clinical application, especially when renal disease is diffuse [61].

Stromal-derived factor (SDF-1) or CXCL12 binds to two receptors, CXCR4 and CXCR7. SDF-1/CXCR4 plays an important role in MSCs [64] and renal progenitor cell [65] migration to damaged kidney. When combined with ultrasound-targeted microbubble destruction (UTMD), a method that increases renal interstitial permeability, exogenous SDF-1 can be released in the kidneys and improve MSC homing [58].

An emerging approach of MSC-based therapies includes the understanding of exosomes role in tissue regeneration. Exosomes are naturally occurring secreted membrane vesicles (30–40 to 100–120 nm) with a ubiquitous presence in biological fluids and an intrinsic homing ability. These extracellular vesicles are considered as important mediators of cell-to-cell communication, mediating the effects of MSCs on target cells, such as transfer of receptors, proteins, and genetic information (mRNA and microRNAs), as well as possessing a direct stimulation on target cells [66]. In preclinical studies, the use of MSC-derived microvesicles is associated with improved organ function following acute injury and may be useful for inhibiting tumor growth [67]. The therapeutic effect of these microvesicles seem to be superior to the effect observed when conditioned-medium is infused in acute injury kidney models [68], although others reported better outcomes with conditioned-medium than with microvesicles in chronic kidney disease in rats [69]. In DN setting, the literature is poor regarding the therapeutic potential of microvesicles. Furthermore, improved preclinical study quality in terms of treatment allocation reporting, randomization, and blinding will accelerate the progress towards clinical trials that should assess feasibility and safety of this therapeutic approach in humans.

On top of that, a key aspect that can adversely affect the therapeutic potential of MSCs is the inflammatory environment at the site of the injury, since it can impact the survival and the engraftment of these cells. Furthermore, anti-inflammatory M2 macrophage-associated cytokines (IL-10, TGF-*β*1, TGF-*β*3, and VEGF) support the growth of MSCs, whereas proinflammatory M1 macrophage-associated cytokines (IL-1*β*, IL-6, TNF-*α*, and IFN-*γ*) inhibit MSC growth in vitro [70]. That observation indicates that the timing of MSC injection is crucial for the success of tissue repair.

Although there is evidence that MSCs can differentiate in vitro into mesangial cells when a coculture system of MSCs and oxidant-injured mesangial cells is established [71], further studies are required to improve our understanding in the crosstalk between MSC-damaged mesangial cells in vivo.

Taking a step forward, since companion or domesticated animals naturally develop many diseases that resemble human conditions, they represent, therefore, a novel source of preclinical models. Several diseases have been reported mainly in dogs, but also in cats, and include the chronic kidney disease (CKD) model. The majority of these studies, although uncontrolled, reported that MSCs are potential candidates for regenerating the damaged tissue, as reviewed by Hoffman and Dow [72].

Furthermore, feline chronic kidney disease (CKD) represents an ideal model to study the impact of drugs and cell therapy to reduce tubule-interstitial fibrosis and glomerulosclerosis, since CKD develops in 80–90% of these animals by age of 15 years. However, ADMSCs (adipose-derived MSCs) injection into cats via intravenous route, 2–4 × 10^6^ cells, repeated three times, was not associated with improvement in renal functional parameters [73, 74]. To note, higher doses of ADMSCs (4 × 10^6^ cells) lead to adverse events during infusion, such as vomiting. Nonetheless, other routes of cell infusion are required to reach definitive conclusions about the therapeutic potential of MSC in feline CKD.

### 4.2. MSC Therapy Humans

The number of registered clinical trials worldwide and MSC-based product Investigational New Drug (IND) submissions to Food and Drug Administration (FDA) have been increasing recently, as well as the diversity in donor and tissue source [75]. Interestingly, the proportion of IND submissions that evaluated BM-MSC-based products was 100% through 2007 but decreased to ~55% by 2012. The number of ADMSC-based product INDs increased significantly since 2011, with a 3-fold increase between 2011 and 2012 alone.

Several types of stem cells have been tested in a wide range of diseases and injuries, mainly in phase I/II trials: human embryonic stem cell-derived retinal pigmented epithelial cells for macular degeneration; human neural stem cells for stroke/cervical spinal cord injury; endothelial stem/progenitor cells for pulmonary arterial hypertension; and placental stem cells for stroke/rheumatoid arthritis/peripheral artery disease, for example, [76].

There is considerable heterogeneity in MSC protocols and a variety of sources used to isolate and manufacture the MSC populations for clinical trials [76]. The majority of the MSC trials are allogeneic cells and these trials are happening in the USA, Europe, and China: phase 1 only (26%), phase 1/2 (40.6%), phase 2 only (22.5%), phase 2/3 (3.8%), phase 3 only (6.7%), and phase 4 only (0.3%). The indications of MSC-based therapy in 352 registered trials comprised bone/cartilage, heart, neurons, immune/autoimmune, diabetes/kidney, lung, liver, and gastrointestinal trials. When type 2 diabetic patients (*n* = 22) were treated with allogeneic Wharton's Jelly-derived MSCs (1 × 10^6^/kg) by both intravenous and intrapancreatic routes, a reduction in glucose and glycated haemoglobin levels associated with decreased systemic inflammation, for example, low levels of IL-1*β* and IL-6, and T-lymphocyte counts (CD3 and CD4) were observed at 12-month follow-up [77]. Likewise, C-peptide levels ameliorated after MSC treatment and insulin requirement decreases by ~50%.

A key aspect of MSC-based therapy is the isolation of MSCs from diabetic individuals for autologous transplant. It is reported that ADMSC from diabetic donors exhibits higher levels of cellular senescence and apoptosis when compared to nondiabetic ADMSC, as well as reduced capacity of osteogenic and chondrogenic differentiation [78]. Hence, allogeneic* versus* autologous MSC-based transplantation requires further investigation in DN setting. In patients with ischemic cardiomyopathy, allogeneic and autologous BM-MSCs were equally safe and effective [79].

Moreover, further characterization of MSC-based manufactured products to better understand the existence, phenotype, and MSC subpopulations is crucial for advancing MSC-based therapies.

In addition, some obstacles need to be overcome in order to provide safety for MSC-based therapies, such as cytogenic aberrations in mice-derived MSC (C57BL/6 and BALB/c) after several passages in vitro [80] and their malignant transformation in vivo either after injection [81] or by promoting growth of a preexisting tumor [82]. For human MSCs, malignant transformation of these cells has not been noted to date in clinical trials [36, 76]. Of importance, beneficial effect of MSCs can be offset by a long-term adipogenic mal-differentiation accompanied by glomerulosclerosis [83].

## 5. Perspectives

**Figure 1:**
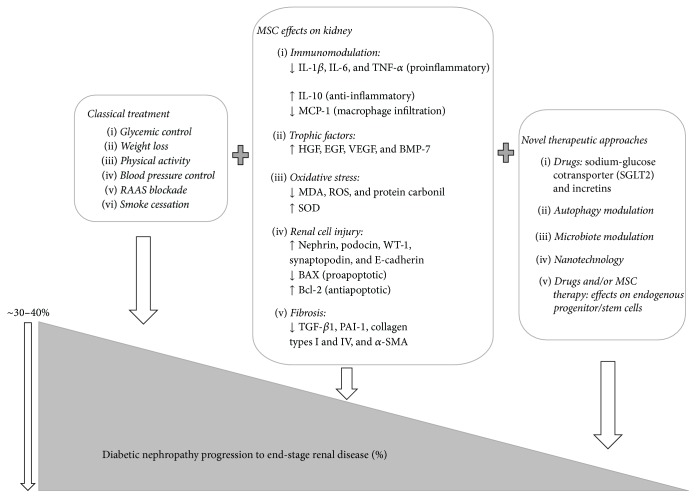
Current treatment to prevent DN, MSC-based therapeutic approaches, and perspectives to halt the progression of DN.

Although there have been major advances in the understanding of the molecular mechanisms that contribute to the development of DN, current best practice still leaves a significant treatment gap. Next, we discuss some perspectives, combined with current available treatment and/or MSC-based approach, that can ultimately contribute to halting the progression of DN, as summarized in Figure 1.

### 5.1. Pharmacologic Therapy

No currently available treatments can prevent the development of diabetic nephropathy. The established therapeutic strategies are mainly based on strict control of glucose levels and blood pressure and blockade of the RAAS. These strategies may slow the progression of renal damage, but many patients still have progressive disease [84]. Although novel agents, such as sulodexide (ameliorates the abnormalities in the glomerular basement membrane and mesangial matrix), pyridoxamine (inhibitor of AGE formation), alagebrium (ACE cross-linker breaker), and ruboxistaurin (inhibitor of PKC-*β*), have been tested in phase II studies, their benefit still requires further investigation [85]. The clinical trial involving aliskiren, a renin inhibitor, was stopped prematurely due to adverse events (hyperkalemia, hypotension, and cardiac arrest) and due to lack of benefit when compared to placebo [86].

A new approach to the management of type 2 DM involves the reduction of renal glucose reabsorption through inhibition of the high-capacity and low-affinity sodium glucose cotransporter (SGLT2), found in the brush border of the first segment of the proximal convoluted tubules [87]. Approximately 160–180 g/day of glucose is filtered and reabsorbed by the kidneys in healthy individuals, reaching ~100–230 g/day with SGLT2 inhibitors [88]. Likewise, urinary glucose excretion is <0.5 g/day in health individuals and increases to ~50–80 g/day with those drugs. SGLT2 inhibitors promote a reduction of 0.59–0.82% in glycated hemoglobin and moderate reduction in body weight (−2.1 to −2.5 kg) and systolic blood pressure (−2.9 to −5.2 mmHg) [89].

Mechanistically, SGLT2 inhibitors (empagliflozin, dapagliflozin, and canagliflozin) reduce proximal tubular sodium reabsorption, thereby increasing distal sodium delivery to the macula densa, which has been shown to activate tubuloglomerular feedback, leading to afferent vasoconstriction and a decrease in the hyperfiltration and intraglomerular pressure [88]. A mild osmotic diuresis also occurs.

The randomized controlled trial EMPA-REG Outcomes with a 3.1-year follow-up documented that empagliflozin leads to significantly lower rates of death from cardiovascular causes (38%), hospitalization for heart failure (35%), and death from any cause (32%) [90]. Empagliflozin was also associated with lower rates of hyperglycemia and lower values for weight and blood pressure, without an increase in cardiac rate, than was placebo.

Regarding renal outcomes in the EMPA-REG trial, empagliflozin was associated significantly with lower rates of incident or worsening nephropathy (12.7% versus 18.8%) [91]. Doubling of the serum creatinine and renal replacement therapy initiation decreased by 44% and 55%, respectively. To note, ~80% of the patients in the empagliflozin and placebo groups were taking standard-of-care drugs (angiotensin-converting enzyme inhibitors or angiotensin-receptor blockers) at baseline, whereas the clearance of creatinine was ~48 mL/min/1.73 m^2^. Urinary albumin-to-creatinine ratio (mg/g creatinine) was < 30 in 46.6%, 30–300 in 33.8%, and >300 in 18.9% and did not change with empagliflozin.

In humans, the only adverse effect was genital and urinary infection in the empagliflozin group [88, 90, 91]. In addition, SGLT2 inhibitors exhibit limited action in patients with severe renal impairment.

In BTBR^*ob*/*ob*^ mice, empagliflozin treatment for 12 weeks, starting at age of 8 weeks, enhanced glycosuria and decreased blood glucose and proteinuria, independently of angiotensin II-induced hypertension [92]. Renal effects were explained by structural improvement (reduction in glomerular tuft area and mesangial expansion) and decreased renal expression of monocyte chemoattractant protein-1 (MCP-1), regulated on activation, normal T cell expressed, and secreted (RANTES) chemokine and IL-6.

In the* db/db* mice, a model of type 2 DN possesses a spontaneous mutation of the leptin receptor and is characterized by polyphagia, obesity, insulin resistance, hyperglycemia, and pancreatic *β*-failure; empagliflozin leads to a decrease in the glomerulosclerosis index and renal expression of TGF-*β*1, without affecting proteinuria, plasmatic cystatin C, and urinary markers (KIM-1 and NGAL) [93]. Tubule-interstitial fibrosis decreased when empagliflozin was associated with metformin.

Glucagon-like peptides 1 (GLP-1) receptor agonists and DPP-4 (dipeptidyl peptidase-4) inhibitors (incretin-based therapies) are also currently available strategies to prevent DN. One of these drugs, liraglutide, can ameliorate kidney fibrosis in a STZ-induced DN model in ED-1 mice by inhibiting TGF-*β*2-mediated endothelial-mesenchymal transition, a key mechanism that has emerged as an important source of matrix-producing fibroblasts [94]. That mechanism seems to be related to microRNA29 induction. Liraglutide can also contribute to decrease proteinuria in STZ-induced DN in Wistar rats by reducing the renal inflammation-mediated by NF-*κ*B, TNF-*α*, MCP-1, IL-6, and INF-*γ* and by increasing eNOS phosphorylation, eNOS activity, and NO production [95]. Likewise, inhibiting DPP4 suppresses renal oxidative stress and receptor for AGE products in the* db/db* mice [96]. Hence, inhibiting DPP-4 may be a therapeutic target for treating kidney fibrosis in diabetes. MSC-based therapy may provide additional benefit in preventing DN when combined with SGLT2 and DPP-4 inhibitors.

### 5.2. Modulation of Autophagy

Podocytes exhibit high levels of constitutive autophagy, a pathway that delivers damaged proteins and organelles to lysosomes, representing a key protective mechanism against podocyte aging and glomerular injury [97]. Therefore, modulating autophagy represents a promising target therapeutic strategy to slow the progression of DN.

Tight balance of mTOR activity is crucial for podocyte homeostasis. mTORC1-Raptor regulates autophagy, whereas mTORC2-Rictor is important for cell survival, metabolism, proliferation, and cytoskeleton maintenance. Genetic deletion of mTORC1 podocyte leads to proteinuria and progressive glomerulosclerosis in mice [98]. That progression is aggravated by simultaneous deletion of mTORC2.

Conversely, mTORC1 is highly activated in podocytes of diabetic mice and patients and may be involved in the mechanisms of diabetes-related autophagy inhibition, which ultimately leads to early glomerular hypertrophy and hyperfiltration in diabetic nephropathy (DN) setting [98]. Hence, when mTORC1 is genetically deleted in podocytes of diabetic mice, the progression of glomerular disease is attenuated [98]. Likewise, SRL, a potent mTORC1 inhibitor, can also ameliorate glomerular lesions in diabetic rat [99]. Furthermore, abnormal mTORC1 activation causes mislocalization of slit diaphragm proteins and induces an epithelial-mesenchymal transition-like phenotypic switch with enhanced endoplasmic reticulum (ER) stress [100]. Collectively, these data indicate that genetic deletion or reduced activity of mTORC1 may protect podocyte and prevent DN-induced glomerulosclerosis.

### 5.3. Fecal Microbiota Modulation

The intestinal microbiota is a complex ecosystem that affects human metabolism and may contribute to the development of obesity, insulin resistance, and subsequent type 2 diabetes. The ability of the intestinal microbiota to affect host metabolism is mediated by at least four components: dietary/nutrients intake, bile acids dehydroxylation, short chain fatty acid (SCFA) metabolism, and gut microbiota composition [101].

Of importance, metagenomic sequencing studies in Chinese and European individuals with type 2 DM indicate that functional alterations of their gut microbiome, for example, dysbiosis, might be directly associated with type 2 DM development [102, 103]. Dysbiosis leads to an increase in lipogenesis in liver and LPS- (lipopolysaccharide-) mediated inflammation in adipose tissue, as well as a decrease in insulin sensitivity and fatty oxidation in muscle [101]. Mechanistically, a reduced amount of butyrate-producing bacteria [102] and the* Akkermansia muciniphila* bacteria [104] is observed in human and mice with type 2 diabetes.

Therapeutic interventions that manipulate the microbiota such as prebiotics, probiotics (live microorganisms), and fecal microbiota transplantation (FMT; infusing intestinal microbiota from lean donor) may have benefits in improving glucose metabolism and insulin resistance in the host [101, 104–108]. FMT in humans with metabolic syndrome has beneficial effects on the recipients' microbiota composition (increase in SCFA-producing bacteria), with a concomitant improvement in insulin sensitivity [109]. These effects may be mediated by normalization of SCFA-producing bacteria. Likewise, FMT from obese to lean mice lead to an increase of 20% in the adiposity [110].

However, it is yet to be proved whether intestinal microbiota plays a causal role in the pathogenesis of obesity and insulin resistance, as well as whether MSC-based therapy can modulate intestinal microbiota towards a less inflammatory environment in DN setting.

### 5.4. Nanotechnology

Nanoparticles possess numerous medical applications and are emerging as a class of carriers for drug and gene delivery.

As previously reported, cell-based strategy utilizing MSCs therapy is very promising for tissue regeneration [111], including DN [35, 58]. MSCs show preferential migration toward sites of inflammation, injury, and cancer, suggesting that these cells may be attractive hypoimmunogenic cellular vehicles for drug and gene delivery [112], as well as trophic factors to damaged diabetic kidney.

The metallic nanoparticles is one of the most utilized, especially due the versatility of this platform, absent immunogenicity, and the ability to allow the cell tracking in vivo by single or multimodal imaging modalities [112–114].

However, understanding the nephrotoxic effect of the nanoparticles is a key aspect to successfully combine that technology with MSC-based therapy. Cytotoxic effects of metallic nanoparticles evaluated in human renal cell lines (IP15, glomerular mesangial cells and HK2, epithelial proximal cells) were mediated by oxidative stress and were associated with metal composition, particle scale, and metal solubility [115]. That cytotoxicity is observed in vitro with different types of nanoparticles (carbon, metal, and/or silica nanoparticles) at both glomerular and tubular levels, such as decreased cell viability, induction of oxidative stress, mitochondrial or cytoskeleton dysfunction, and cell membrane and DNA damage [116].

Nanoparticle diameter is also a crucial aspect for targeting kidney tissue. Therefore, gold-based nanoparticles of ~75 ± 25 nm may target the mesangium of the kidneys [117]. Of importance, MSCs transfected with nanoparticles of that size may provide a novel strategy to target mesangial cells in DN, yet mesangial expansion is the hallmark of early stages of DN. Moreover, designing nanoparticles to target the kidney should take into account the notion of renal clearance and glomerular filtration, because small nanoparticles (<10 nm) can be filtered through glomerular filtration and be lost in the urine. On the other hand, prolonged retention of the nanoparticles inside the kidneys can be toxic due to excessive nanoparticle uptake by renal cells.

These new insights utilizing MSC therapy combined with nanoparticles are appealing and open new possibilities for the treatment of injured renal cells in DN setting.

### 5.5. Kidney-Derived Stem/Progenitor Cells

Although exogenous MSCs exhibit therapeutic potential, endogenous MSCs do not migrate to damaged kidneys [118]. However, postnatal tissues have reservoirs of specific progenitor/stem cells, which contribute to maintenance and regeneration [119].

In human adult kidney, a hierarchical CD24^+^CD133^+^ population of progenitors cells organized in a precise sequence along Bowman's capsule (PECs, parietal epithelial cells) was identified as a reservoir of cells that may contribute to the turnover of senesced or injured podocytes by proliferating, migrating, and differentiating from the urinary to the vascular stalk [120]. However, lineage tracing studies documented that their recruitment occurs mainly in juvenile mice [121] and only a small fraction of these cells are recruited to glomeruli in adult mice [122]. Likewise, PECs were not involved in podocyte regeneration in models of glomerular hypertrophy in adult animals [122], although these cells were identified as a possible source of regenerating podocytes after treatment with glycogen synthase kinase 3-*α* and 3-*β* (GSK3s) inhibitor [123]. Of importance, abnormal proliferation of these renal progenitors of the Bowman's capsule can generate hyperplastic glomerular lesions and scarring in collapsing glomerulopathy and crescentic glomerulonephritis [124].

The leptin-deficient BTBR^*ob*/*ob*^ mouse provides a model of advanced but reversible DN. Furthermore, leptin replacement resulted in near-complete reversal of functional and structural measures of advanced DN [125]. Hence, proteinuria and accumulation of reactive oxygen species ameliorate when leptin was administered, as well as the morphological-related DN lesions, such as mesangial matrix expansion, mesangiolysis, GBM thickening, and podocyte loss. To note, PECs (identified with the podocyte markers Wilms tumor 1 and p57) contributed to generating new podocytes. On top of that, inhibition of the renin-angiotensin-aldosterone system (RAAS) did not reverse DN-related lesions, which can at least in part explain the limited efficacy of RAAS inhibitors in promoting repair of DN.

In addition, since paracrine factors have been proposed as a key mechanism of benefit of MSC cell therapy, some studies pointed out that MSC may stimulate endogenous progenitor/stem cell proliferation and differentiation and therefore contribute to tissue repair [126], although these effects were not observed by others [127].

Understanding why MSCs have the potential to stimulate endogenous progenitor/stem cells may enable the future development of pharmacoregenerative therapies, as well as improving current cell therapy strategies. If MSCs possess the capacity to stimulate kidney-derived progenitor/stem cells [128], including the recent c-Kit^+^ cell population described recently by our group [129], further experiments in different experimental models and in human tissue will be necessary for a definitive picture of MSC in kidney regeneration.

### 5.6. MSC-Derived from Induced Pluripotent Stem Cells (iPSCs)

Induced pluripotent stem cells (iPSCs) are generated from somatic cells and represent a potentially inexhaustible cell resource with a pluripotent potential similar to embryonic stem cells. Functional MSCs derived from iPSCs possess similar mesenchymal characteristics of the naïve BM-MSCs, such as positivity for typical mesenchymal markers and negativity for endothelial and hematopoietic markers, as well as trilineage differentiation properties [130]. Of importance, these iPSC-MSCs exhibit therapeutic potential in models of limb ischemia [131] and diabetic polyneuropathy [132]. However, their regenerative capacity during DN development requires further studies.

## 6. Conclusions

MSCs have several advantages for therapeutic purposes, such as their ability to migrate to injured tissues, strong immunosuppressive effects, safety profile, and lack of ethical issues, such as those related to the application of human embryonic stem cells. Therefore, MSC-based therapy is expected to become a promising strategy to slow DN progression because of their robust paracrine effects. Moreover, MSCs-based therapy combined with new drugs and/or novel therapeutic approaches, such as the modulation of fecal microbiota and renal autophagy, and the design of nanoparticles to enhance MSC effects will provide insightful strategies to prevent DN. In addition, the better understanding of the crosstalk between MSC and resident progenitor/stem cells may unveil a new mechanism of MSC therapy.

Despite the similarities between the sources of MSCs have already been documented, some important differences should be taken into account when choosing the MSC source for research or therapeutic purposes. Whether allogeneic and autologous MSC-based therapies harbor the same potential to treat kidney fibrosis in DN, there will be many MSC products that will meet the criteria for registered products in the established regulatory systems over the next years

To note, whether those findings in animals models will translate into reduced proteinuria and glycemia in humans with DN can only be determined in adequately powered, randomized, and controlled trials.

## Figures and Tables

**Table 1:** Preclinical studies in rodents to test the potential of MSCs in DN.

MSC isolation/type of transplant	Model of DN and groups	Number of injections/route of delivery	Number of cells injected	Results	Reference
h-BM-MSCs, xenotransplant	STZ-induced type 1 NOD/*scid* mice: normal, DN, DN + hMSC	Single dose, intracardiac	2.5 × 10^6^	DN + hMSC versus DN: ↑ pancreatic insulin content and islet cell number ↓ renal macrophage infiltration Improvement in renal histology	[45]

BM-MSCs, allogeneic	STZ-induced type 1 diabetes C57BL/6 mice: DN + vehicle and DN + MSC	Single dose, IV	0.5 × 10^6^	DN + MSCs versus DN: ↓ blood glucose levels ↓ albuminuria and glycosuria Improvement in renal and *β*-cell histology	[46]

BM-MSCs, allogeneic	STZ-induced type 1 diabetes C57BL/6 mice: Control, DN + vehicle, DN + MSC	Two doses (interval of 20 days), IV	0.5 × 10^6^	DN + MSCs versus DN: ↓ albuminuria Improvement in renal histology No improvement in *β*-cell function and histology	[47]

BM-MSCs, allogeneic	STZ-induced type 1 diabetes Sprague-Dawley Rats: DN control, MSC, CSA, MSC + CSA (MSCA)	single dose, intracardiac	2 × 10^6^	MSCA group versus DN: ↓ blood glucose levels ↓ albuminuria Improvement in renal mass index	[48]

ADMSCs, autologous	STZ-induced type 1 diabetes Sprague-Dawley Rats: Control non-diabetic, DN + vehicle, ADMSC + DN	Single dose, IV	1 × 10^7^	DN + ADMSCs versus vehicle: ↓ renal p-p-38, p-ERK and p-JNK ↓ renal MDA and carbonyl protein ↓ renal TNF-*α*, IL-1*β*, IL-6 ↓ renal MnSOD and CuZn-SOD	[49]

h-UCB-SCs, xenotransplant	STZ-induced type 1 diabetes Sprague-Dawley Rats: control, DN, DN + h-UCB-SC	Single dose, IV	1 × 10^6^	DN + h-UCB-SCs versus DN: ↓ blood glucose levels ↓ albuminuria ↓ renal fibronectin, *α*-SMA ↑ renal E-cadherin	[50]

h-UCB-SCs, xenotransplant	STZ-induced type 1 diabetes Sprague-Dawley Rats: control, DN, DN + h-UCB-SC	Single dose, IV	5 × 10^5^	DN + h-UCB-SCs versus DN: *↔* blood glucose levels *↔* albuminuria Improvement in renal histology ↓ renal TGF-*β*1, *α*-SMA ↑ renal E-cadherin, BMP-7	[51]

BM-MSCs, allogeneic	STZ-induced type 1 diabetes Sprague-Dawley Rats: Normal control, DN + MSC and DN + medium	Single dose, left renal artery	2 × 10^6^	DN + MSCs versus DN + medium: *↔* blood glucose levels ↓ kidney weight, kidney/body weight, creatinine clearance ↓ albuminuria Improvement in renal histology ↑ renal nephrin, podocin, VEGF, BMP-7	[52]

BM-MSCs, allogeneic, UTDM	STZ-induced type 1 diabetes Sprague-Dawley Rats: Normal control, DN + PBS, DN + UTMD, DN + MSC, DN + MSC + UTMD	Single dose, IV	1 × 10^6^	MSC and MSC + UTMD versus DN and UTMD: ↓ blood glucose levels ↑ plasma insulin Attenuated *β*-cell damage ↓ albuminuria ↓ renal TGF-*β*1 ↑ renal synaptopodin, IL-10 After UTMD: MSC homing was increased to kidneys (~2x)	[53]

BM-MSCs, allogeneic	STZ-induced type 1 diabetes Wistar Rats: Normal control, DN + vehicle, DN + MSC	2 doses (1 week a part), IV	2 × 10^6^	DN + MSCs versus DN: ↓ blood glucose levels ↓ albuminuria ↓ creatinine clearance Improvement in renal histology ↓ renal MCP-1, ED-1, IL-1*β*, IL-6, TNF-*α* ↑ renal HGF	[54]

BM-MSCs, allogeneic	STZ-induced type 1 diabetes Wistar rats: DN, DN + MSC, DN + Insulin, DN + Probucol	2 doses (1 week a part), IV	2 × 10^6^	DN + MSCs versus DN: ↓ blood glucose levels ↓ albuminuria ↓ creatinine clearance ↓ kidney/body weight Improvement in renal histology ↓ renal fibronectin and collagen I ↓ renal MDA content ↓ renal TGF-*β*1 ↓ renal ROS fluorescence ↑ renal SOD activity ↓ cellular glucose uptake mediated by GLUT1 in kidneys	[55]

BM-MSCs, allogeneic	STZ-induced type 1 diabetes albino rats: Control, DN, DN + PBS, DN + MSC	Single dose, IV	1 × 10^6^	DN + MSCs versus DN: ↓ blood glucose levels ↓ albuminuria ↓ body weight ↓ serum creatinine and urea ↑ renal VEGF and anti-apoptotic bcl2 ↓ renal TNF-*α*, pro-apoptotic Bax, TGF-*β* Improvement in renal histology	[56]

BM-MSCs, allogeneic	Normal control, DN + saline, DN + MSC	2 doses (1 week a part), IV	2 × 10^6^	DN + MSCs versus DN: ↓ blood glucose levels ↓ albuminuria ↓ kidney/body weight ↓ creatinine clearance Improvement in renal histology ↓ renal collagen I, collagen IV, *α*-SMA, TGF-*β*, P-smad3/smad2/3 ↑ renal E-cadherin, BMP7	[57]

BM-MSCs, allogeneic SDF-1-loaded microbubbles	STZ-induced type 1 diabetes Sprague-Dawley Rats: Control, UTMD, UTMD + MSC	Single dose, IV	1 × 10^6^	Improvement in renal histology	[58]

BM-MSCs, allogeneic	STZ-induced type 1 diabetes C57BL/6 mice: DN + vehicle, DN + MSC	Single dose, IV	0.5 × 10^6^	DN + MSCs versus DN: ↓ kidney ↓ kidney/body weight ↓ serum creatinine, urea, and plasma cystatin C ↓ renal collagen I and fibronectin ↓ renal tubular apoptotic index, ROS total, lipid peroxidation, oxidative protein damage, F4/80 positive cells ↑ renal nephrin, tubular Ki67 proliferation index ↑ plasma bFGF, EGF, HGF, IL-6, and IL-10 Improvement in renal histology	[59]

BM-MSCs, allogeneic	STZ-induced type 1 diabetes Sprague-Dawley rats: control, DN, DN + MSC	Single dose, IV	2 × 10^6^	MSCs + DN versus DN: *↔* blood glucose levels ↓ albuminuria ↓ kidney weight ↓ serum creatinine ↓ renal PAI-1, TGF-*β*1, Smad3	[60]

MSCs: mesenchymal stem cells; BM-MSC: bone marrow-derived MSCs; h-BM-MSC: human bone marrow-derived MSC; ADMSC: adipose-derived MSCs; h-UCB-SCs: human umbilical cord blood-derived stem cells; DN: diabetic nephropathy; STZ: streptozotocin; CSA: cyclosporine; PBS: phosphate buffered saline; IV: intravenous; *α*-SMA: *α*-smooth muscle actin; bFGF: basic fibroblast growth factor; BMP-7: bone morphogenic protein-7; EGF: epidermal growth factor; HGF: hepatocyte growth factor; MCP-1: monocyte chemoattractant protein-1; SDF-1: stromal derived factor-1; TGF-*β*: transforming growth factor *β*; TNF-*α*: tumor necrosis factor-*α*; VEGF: vascular endothelial growth factor; IL: interleukin; SOD: superoxide dismutase; MDA: malondialdehyde; ROS: reactive oxygen species; UTMD: ultrasound-targeted microbubble destruction; PAI-1: plasminogen activator inhibitor-1.

## Abbreviations

ADMSC: Adipose-derived MSCs
AGEs: Advanced glycosylation end products
AMDCC: Animal Models of Diabetic Complications Consortium
BM-MSC: Bone marrow-derived MSCs
BTBR^*ob*/*ob*^ mice: Black and tan, obese, and tufted ob/ob (leptin deficient) mice
CKD: Chronic kidney disease
DCs: Dendritic cells
DM: Diabetes mellitus
DN: Diabetic nephropathy
DPP-4 inhibitors: Dipeptidyl peptidase-4 inhibitors
eNOS: Endothelial nitric oxide synthase
FMT: Fecal microbiota transplantation
GBM: Glomerular basement membrane
HGF: Hepatocyte growth factor
IL: Interleukin
IND: Investigational New Drug
IFN-*γ*: Interferon-*γ*
LPS: Lipopolysaccharide
MHC: Major Complex of Histocompatibility
MCP-1: Monocyte chemoattractant protein-1
MSCs: Mesenchymal stem cells
NK cells: Natural killer cells
NOD: Nonobese diabetic mouse
PECs: Parietal epithelial cells
PKC: Protein kinase C
RAAS: Renin-angiotensin-aldosterone system
SCFA: Short chain fatty acid
SGLTs: Sodium-glucose co-transporters
STZ: Streptozotocin
TGF-*β*1: Transforming growth factor-*β*1
TNF-*α*: Tumor necrosis factor-*α*
UAE: Urinary albumin excretion
UCB-MSCs: Umbilical cord blood-derived MSCs
UTMD: Ultrasound-targeted microbubble destruction
VEGF-A: Vascular endothelial growth factor A.
